# Application of Fatty Liver Inhibition of Progression Algorithm and Steatosis, Activity, and Fibrosis Score to Assess the Impact of Non-Alcoholic Fatty Liver on Untreated Chronic Hepatitis B Patients

**DOI:** 10.3389/fcimb.2021.733348

**Published:** 2022-01-17

**Authors:** Yan Huang, Qinyi Gan, Rongtao Lai, Weijing Wang, Simin Guo, Zike Sheng, Lu Chen, Qing Guo, Wei Cai, Hui Wang, Gangde Zhao, Zhujun Cao, Qing Xie

**Affiliations:** Department of Infectious Diseases, Ruijin Hospital, Shanghai Jiao Tong University School of Medicine, Shanghai, China

**Keywords:** hepatitis B, non-alcoholic fatty liver disease (NAFLD), non-alcoholic steatohepatitis (NASH), liver biopsy, liver fibrosis

## Abstract

**Backgrounds and Purpose:**

Concurrent non-alcoholic fatty liver disease (NAFLD) in chronic hepatitis B (CHB) patients is a frequent and increasingly concerning problem because of the NAFLD pandemic. Admittedly, NAFLD can progress to non-alcoholic steatohepatitis (NASH) and severe fibrosis. Direct evidence of the fibrotic effect of NAFLD or NASH in chronic hepatitis B virus (HBV) infection remains lacking. We aimed to reveal the influence of concurrent histologically proven fatty liver diseases in fibrogenesis with chronic HBV infection.

**Methods:**

We performed a retrospective cross-sectional study on a liver biopsy population of CHB patients without excessive alcohol intake to evaluate the prevalence of concurrent histologically proven NAFLD or NASH according to the fatty liver inhibition of progression (FLIP) algorithm and its association with the liver fibrosis stage.

**Results:**

Among 1,081 CHB patients, concurrent NAFLD was found in 404 patients (37.4%), among whom 24.0% (97/404) have NASH. The presence of NASH was an independent predictor of significant fibrosis (odds ratio (OR), 2.53; 95% CI, 1.52–4.21; *p* < 0.001) and severe fibrosis (OR, 1.83; 95% CI, 1.09–3.09; *p* = 0.023) in all patients, as well as in patients with normal alanine aminotransferase (ALT) (predicting significant fibrosis, OR, 2.86, 95% CI, 1.34–6.10; *p* = 0.007). The presence of lobular inflammation (*p* < 0.001) or presence of cytological ballooning (*p* < 0.001), rather than presence of steatosis (*p* = 0.419), was related with severity of fibrosis in Spearman’s correlation analysis.

**Conclusions:**

Concurrent NAFLD is common in CHB patients, and NASH is an independent risk factor potentiating significant fibrosis by 2.53-fold and severe fibrosis by 1.83-fold. While coping with chronic HBV infection, routine assessment of co-existing NAFLD or NASH is also important.

## Introduction

As a major global public health problem, chronic hepatitis B (CHB) affects an estimated 292 million people (Ott et al., 2017; Polaris Observatory, 2018). With the increased incidence of obesity and metabolic syndrome, non-alcoholic fatty liver (NAFL) disease (NAFLD) has become another important cause of liver cirrhosis and hepatocellular carcinoma (HCC) worldwide, affecting roughly 25% of the world’s population (Loomba and Sanyal, 2013; Younossi et al., 2016; Younossi et al., 2018). The disease spectrum of NAFLD ranges from NAFL to non-alcoholic steatohepatitis (NASH), which is histologically characterized by the presence of steatosis, inflammation, and ballooning. Because of their high prevalence and increased diagnosis of NAFLD, superimposed NAFLD in chronic hepatitis B virus (HBV) infection has been increasingly common in clinical practice nowadays.

It is reasonable to suppose that the development of NAFLD in CHB would exaggerate liver lesions and accelerate the remodeling of liver structure, leading to an increased risk of disease progression.

Previous studies have demonstrated that metabolic syndrome is an independent risk factor for fibrosis progression (Wong et al., 2009; Adams et al., 2017), cirrhosis, and poor clinical outcomes (Cheng et al., 2016) in CHB patients. A large multicenter cohort study revealed that patients with concomitant NASH and CHB have a higher risk of developing liver-related outcomes (Choi et al., 2020). However, several studies suggested that hepatic steatosis in CHB patients does not correlate with the activity of necroinflammation or stage of liver fibrosis (Thomopoulos et al., 2006; Bondini et al., 2007; Zheng et al., 2010). The impact of steatosis or steatohepatitis on disease progression remains inconclusive in these studies. Our hypothesis was that the presence of NASH in CHB patients was associated with an increased risk of significant and severe fibrosis.

The current study was aimed to test this hypothesis in a large cohort of CHB patients with liver biopsy for histological assessment.

## Materials and Methods

### Study Design

This is a cross-sectional analysis of a prospectively collected dataset for patients receiving percutaneous liver biopsy in Shanghai Ruijin Hospital. Details of the biopsy procedure, data collection, and biobank maintenance were described previously (Cao et al., 2017a; Cao et al., 2017b). All the patients provided written informed consent for liver biopsy. The indications for liver biopsies are as follows: 1) staging of known parenchymal liver disease to predict prognosis; 2) instructing on whether to start antiviral therapy based on the histologic staging of inflammation and fibrosis, especially in patients with normal or mildly elevated alanine aminotransferase (ALT); and 3) diagnosis of other liver diseases other than HBV infection. Currently, there is no established scoring system for assessing the activity of fatty liver in patients with CHB. For the purpose of analysis, a robust score, “steatosis, activity, and fibrosis (SAF) score” established (Bedossa and Consortium, 2014) and validated (Nascimbeni et al., 2020) in patients with NAFLD was used to evaluate steatosis, lobular inflammation, and ballooning degeneration. Based on the SAF score, patients were classified into three groups—NASH, NAFL, and no NAFLD—according to the fatty liver inhibition of progression (FLIP) algorithm. All the liver specimens from eligible patients were retrospectively assessed for the SAF score and FLIP classification. The current study complied with the Declaration of Helsinki and was approved by the ethics review committee of Shanghai Ruijin Hospital. Patient informed consent was waived by the committee considering the retrospective design.

### Patients

All patients who received liver biopsy between January 2009 and December 2018 at our unit were assessed for eligibility. Inclusion criteria i) aged older than 16 years and ii) with positive serum hepatitis B surface antigen for at least 6 months. Exclusion criteria were i) with evidence of other chronic liver diseases including other viral hepatitis, autoimmune hepatitis, drug-induced liver disease, and primary biliary cholangitis; ii) with evidence of HCC; iii) with previous history of antiviral therapy; iv) with unreliable scoring results derived from unqualified liver samples, defined as less than 10 mm or containing less than six portal triads; and v) with excessive alcohol assumption, defined as alcohol intake of ≥20 g per day for men and ≥10 g per day for women. A flowchart of the study design is illustrated in **Supplementary Figure 1**.

### Clinical Evaluation

All patients received comprehensive clinical and laboratory assessments within 1 month before liver biopsy. Medical history of hypertension, diabetes mellitus (DM), alcohol consumption, and anthropometric parameters including body weight and body height were recorded. Overweight was defined as body mass index (BMI) ≥23 kg/m^2^, and obesity as BMI ≥ 25 kg/m^2^, following Asian-specific recommendations (Consultation, 2004). The upper limit of normal (ULN) for ALT was defined as ≤40 U/L according to the European Association for the Study of the Liver (EASL) criteria (European Association for the Study of the Liver. Electronic address and European Association for the Study of the, 2017).

### Histological Evaluation

Biopsy specimens were routinely stained with H&E and Masson trichrome stains and assessed by two liver pathologists immediately after liver biopsies. Scheuer’s scoring system was used for staging fibrosis (S0–S4) and inflammation (G0–G4) (Scheuer, 1991). Significant fibrosis was defined as S ≥ 2 and severe fibrosis as S ≥ 3. Steatosis, lobular inflammation, and cytological ballooning degeneration were reevaluated according to the SAF score by a liver pathologist blinded to clinical data/biological data. According to the SAF scoring system, steatosis was scored on a 0–3 scale, lobular inflammation on a 0–2 scale, and ballooning on a 0–2 scale (Bedossa and Consortium, 2014). Histopathological lesions in NAFLD were assessed separately in the SAF score so that the SAF score was recommended and adopted to assess NAFLD in this article (Nascimbeni et al., 2020). Moderate-to-severe steatosis was defined as intrahepatic steatosis ≥33%. Liver specimens with a length ≥10 mm or containing at least six portal triads were considered qualified for analysis, and the rest were excluded.

### Statistical Analysis

Continuous variables were expressed in mean ± SD or median (interquartile range [IQR]), as appropriate, whereas categorical variables were presented as number (percentage). Differences in continuous variables were examined for statistical significance using Student’s t-test or Kruskal–Wallis rank-sum test depending on data distribution. Categorical variables were analyzed with the chi-squared test or Fisher’s exact test. Correlation analyses were performed according to Spearman’s method. Logistic regression analyses were used to identify risk factors for significant fibrosis or severe fibrosis. All the variables with a *p*-value less than 0.1 under univariate analysis entered the stepwise selection process, and those with a *p*-value less than 0.05 were retained. Variables were expressed as odds ratio (OR) and 95% CI. *p*-Values <0.05 were considered statistically significant. We classified our patients into lean or non-lean groups according to the ethnic-specific BMI cutoff of 23 kg/m^2^ in our current study with the Asian population. Subgroup analyses were performed by logistic regression. All data were analyzed using SAS (Version 9.4; SAS Institute Inc., Cary, NC, USA).

## Results

### Characteristics of the Study Population

A total of 1,081 patients with treatment-naïve CHB were identified from 1,701 CHB patients who underwent a liver biopsy between 2009 and 2018 (**Supplementary Figure 1**), and patient characteristics are shown in **Table 1**. Among 1,081 treatment-naïve CHB patients, the median age was 37 years (IQR, 31–47), and 63.1% of them were males. Their median viral load was 5.33 log10 IU/ml (IQR, 3.74–7.18 log10 IU/ml), and 44.4% of them were HBeAg-negative patients. The overall prevalence of diabetes, hypertension, and overweight was 1.5%, 3.1%, and 50.8%, respectively. ALT elevation in this population was ubiquitous and accounted for 52.0% of all patients. The percentage of significant fibrosis (S ≥ 2) and severe fibrosis (S ≥ 3) according to Scheuer’s scoring system was 50.1% and 22.9%, respectively. The histopathological findings are illustrated in detail in **Table 1**.

**Table 1:** Demographic, clinical, and biochemical characteristics and liver pathological features of the patient population.

Variable	All (n = 1,081)	No NAFLD (n = 677)	NAFL (n = 307)	NASH (n = 97)	*p*-Value^1^
Age (years)	37 (31–47)	37 (30–46)	38 (31–47)	42 (35–50)	<0.001
Male (n, %)	682 (63.1%)	387 (57.2%)	222 (72.3%)	62 (77.5%)	<0.001
BMI (kg/m^2^)	23.0 (20.8–25.3)	22.3 (20.3–24.2)	23.9 (21.9–26.1)	25.5 (23.5–27.7)	<0.001
<23 (normal) (n, %)	512 (49.2%)	390 (59.0%)	104 (36.8%)	18 (18.6%)	<0.001
23–24.9 (overweight) (n, %)	430 (41.3%)	239 (36.2%)	139 (49.1%)	52 (53.6%)	
≥25 (obese) (n, %)	99 (9.5%)	32 (4.8%)	40 (14.1%)	27 (27.8%)	
Diabetes (n, %)	16 (1.5%)	5 (0.7%)	7 (2.3%)	4 (4.1%)	0.004
Arterial hypertension (n, %)	33 (3.1%)	14 (2.1%)	8 (2.6%)	11 (11.3%)	<0.001
Family HCC history (n, %)	124 (11.5%)	69 (10.2%)	41 (13.4%)	83 (85.6%)	0.093
Platelets (10^9^/L)	172 (142–206)	172 (143–202)	168 (140–208)	186 (147–216)	0.242
Albumin (g/L)	43 (28–73)	44 (41–46)	43 (40–45)	45 (41–47)	0.005
TB (μmol/L)	16 (12–20)	16 (12–20)	17 (13–22)	14 (11–18)	0.001
ALT (IU/L)	42 (28–73)	40 (26–71)	45 (31–78.5)	43 (28–62)	0.008
ALT > 40 IU/L (n, %)	528 (52.0)	314 (48.9%)	162 (57.9%)	52 (55.3%)	0.035
AST (IU/L)	34 (26–48)	33 (25–48)	35 (27–52)	32.5 (26–42)	0.117
HBsAg (log IU/ml)	3.51 (3.00–4.17)	3.51 (2.98–4.16)	3.52 (3.01–4.34)	3.43 (3.13–4.01)	0.890
HBeAg-positive (n, %)	582 (55.6%)	368 (55.8%)	177 (61.0%)	37 (38.1%)	0.084
HBV DNA (log IU/ml)	5.34 (3.72–7.18)	5.40 (3.74–7.25)	5.57 (3.92–7.15)	4.38 (3.31–5.98)	0.002
HBV genotype, n (%)					
B	101 (34.0%)	68 (36.2%)	25 (29.1%)	8 (34.8%)	
C	188 (63.3%)	119 (63.3%)	54 (62.8%)	15 (65.2%)	0.355
Other	8 (2.7%)	1 (0.5%)	7 (8.1%)	0 (0.0%)	
Significant fibrosis (n, %)	542 (50.1%)	351 (51.9%)	119 (38.8%)	72 (74.2%)	0.273
Severe fibrosis (n, %)	247 (22.9%)	137 (20.2%)	76 (24.8%)	34 (35.1%)	0.001
Steatosis (n, %)					
0, <5%	677 (62.6%)	677 (100.0%)	0 (0%)	0 (0%)	<0.001
1, 5%–33%	308 (28.5%)	0 (0%)	239 (77.9%)	69 (71.1%)	
2, 33%–66%	79 (7.3%)	0 (0%)	54 (17.6%)	25 (25.8%)	
3, ≥66%	17 (1.6%)	0 (0%)	14 (4.6%)	3 (3.1%)	
Lobular inflammation (n, %)					
0, no foci	332 (30.7%)	193 (28.5%)	139 (45.3%)	0 (0%)	0.172
1, <2 foci	669 (61.9%)	435 (64.3%)	145 (47.2%)	89 (91.2%)	
2, ≥2 foci	80 (7.4%)	49 (7.2%)	23 (7.5%)	8 (8.3%)	
Cytological ballooning (n, %)					
0, none	884 (81.8%)	582 (86.0%)	302 (98.4%)	0 (0%)	<0.001
1, few	186 (17.2%)	93 (13.7%)	5 (1.6%)	88 (90.7%)	
2, many	11 (1.02%)	2 (0.3%)	0 (0%)	9 (9.3%)	

Data are presented as mean ± SD, median (IQR), or number of patients (%). Fibrosis was assessed according to Scheuer’s scoring system, in which significant fibrosis was S ≥ 2 and severe fibrosis was S ≥ 3.

BMI, body mass index; TB, total bilirubin; ALT, alanine aminotransferase; AST, aspartate aminotransferase; HBsAg, hepatitis B surface antigen; HBeAg, hepatitis B e-antigen; IQR, interquartile range; NAFLD, non-alcoholic fatty liver disease; NAFL, non-alcoholic fatty liver; NASH, non-alcoholic steatohepatitis; HCC, hepatocellular carcinoma; HBV, hepatitis B virus.

^1^p-Value for comparison between 3 groups of no NAFLD, NAFL, and NASH.

**Figure 1:**
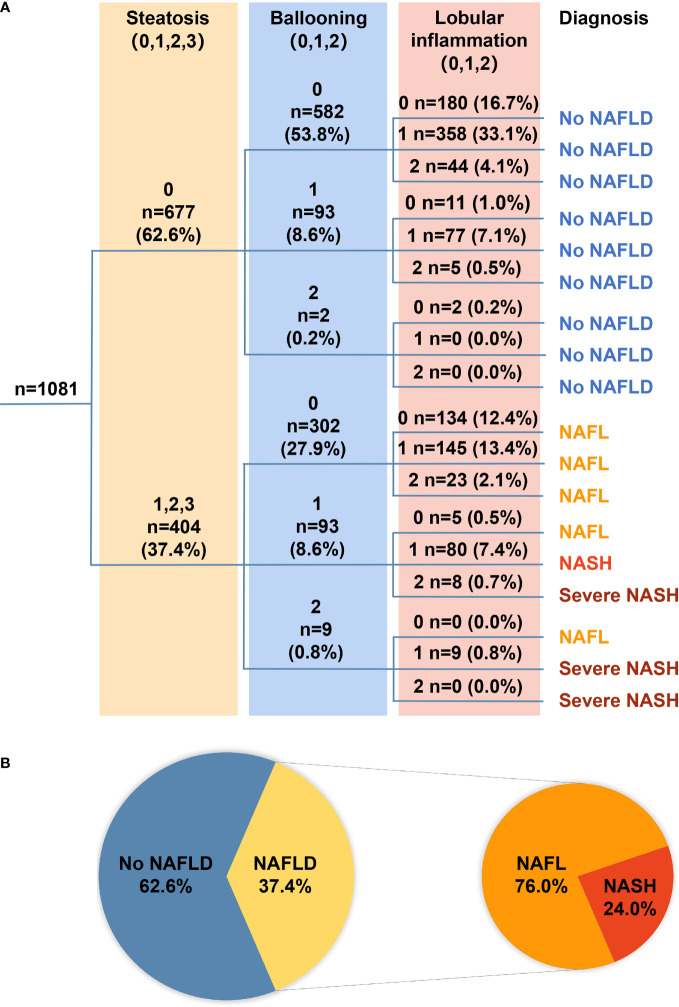
**(A)** The FLIP algorithmic tree. A total of 1,081 patients with treatment-naïve chronic hepatitis B were classified into no NAFLD, NAFL, NASH, or severe NASH according to the FLIP algorithmic pathways based on the grade of steatosis [**(A)**; light yellow shade], grade of ballooning [**(B)**; light blue shade], and grade of lobular inflammation [**(A)**; pink shade]. Number of patients and the proportion among each category were presented as number (%). **(B)** Pie chart illustrates the proportion of patients with specific diagnoses. Among all 1,081 patients, 62.6% had no NAFLD, and the remaining 37.4% had NAFLD, among whom 76.0% had NAFL and 24.0% had NASH. FLIP, fatty liver inhibition of progression; NAFLD, non-alcoholic fatty liver disease; NAFL, non-alcoholic fatty liver; NASH, non-alcoholic steatosis hepatitis.

The FLIP algorithmic tree shows the number of patients in the different algorithmic pathways (**Figure 1A**). According to the FLIP algorithm, 62.6% (677/1,081), 28.4% (307/1,081), and 9.0% (97/1,081) CHB patients were classified as without NAFLD, with NAFL, and with NASH, respectively. Among all the patients with NAFLD, about a quarter (24.0%, n = 97) of patients were diagnosed with NASH, among whom 17.5% (17 of 97) patients were diagnosed with severe NASH (**Figure 1B**).

Compared with patients without NAFLD, patients with NAFL or NASH showed significantly higher median age, BMI, and ALT (all *p* < 0.01) (**Table 1**). A higher proportion of male, diabetic, and arterial hypertensive patients were observed in patients with NAFLD than in those without (all *p* < 0.01) (**Table 1**).

### Association Between Steatohepatitis and Severity of Liver Fibrosis

The proportion of different fibrosis stages between NAFL and NASH subgroups was different (**Table 1**). Percentage of patients with significant fibrosis or severe fibrosis was significantly higher in NASH subgroups than those in NAFL subgroups (74.2% vs. 38.8%, *p* < 0.001, or 35.1% vs. 24.8%, *p* = 0.047, respectively) (**Table 1**).

Factors associated with significant fibrosis or severe fibrosis are shown in **Table 2**. The presence of NASH, overweight, age, blood platelets count, ALT, hepatitis B surface antigen level, HBeAg status, and serum HBV DNA level were significantly associated with the presence of significant fibrosis (**Table 2**). Among these factors, the presence of NASH, overweight, age, and blood platelets count were independent predictors for significant fibrosis under a multivariable analysis. Using the same analytic strategy, we identified the presence of NASH, diabetes, overweight, blood platelets count, albumin, and HBV DNA level as independent factors associated with severe fibrosis (**Table 2**).

**Table 2:** Factors associated with significant fibrosis and severe fibrosis among patients with chronic HBV infection.

Variable	Significant fibrosis (S ≥ 2)				Severe fibrosis (S ≥ 3)			
	Univariable analysis		Multivariable analysis		Univariable analysis		Multivariable analysis	
	OR (95% CI)	*p*-Value	OR (95% CI)	*p*-Value	OR (95% CI)	*p*-Value	OR (95% CI)	*p*-Value
Age (years)	1.03 (1.01–1.04)	<0.001	1.02 (1.01–1.03)	0.004	1.04 (1.02–1.05)	<0.001		
Male (yes/no)	1.17 (0.92–1.50)	0.205			1.18 (0.87–1.59)	0.282		
Diabetes (yes/no)	1.67 (0.60–4.63)	0.324			4.47 (1.65–12.12)	0.003	4.10 (1.14–14.74)	0.031
Hypertension (yes/no)	1.55 (0.76–3.15)	0.225			1.08 (0.48–2.43)	0.847		
Overweight (yes/no)	1.66 (1.30–2.12)	<0.001	1.50 (1.13–1.99)	0.005	1.82 (1.36–2.44)	<0.001	1.80 (1.27–2.54)	<0.001
Family HCC history (yes/no)	1.08 (0.74–1.57)	0.679			1.07 (0.68–1.69)	0.782		
Platelets (10^9^/L)	0.99 (0.99–0.99)	<0.001	0.99 (0.99–1.00)	<0.001	0.98 (0.98–0.99)	<0.001	0.98 (0.98–0.99)	<0.001
Albumin (g/L)	0.99 (0.96–1.03)	0.687			0.92 (0.88–0.95)	<0.001	0.93 (0.89–0.97)	0.002
TB (μmol/L)	1.04 (0.99–1.02)	0.387			1.00 (0.99–1.01)	0.854		
ALT (IU/L)	1.00 (1.00–1.00)	0.046			1.00 (1.00–1.00)	0.242		
AST (IU/L)	1.00 (1.00–1.00)	0.823			1.00 (1.00–1.00)	0.539		
HBsAg (log IU/ml)	0.80 (0.68–0.95)	0.009			0.78 (0.65–0.93)	0.006		
HBeAg-positive (yes/no)	0.76 (0.59–0.97)	0.025			0.76 (0.57–1.02)	0.068		
HBV DNA (log IU/ml)	0.85 (0.82–0.93)	<0.001			0.86 (0.80–0.93)	<0.001	0.88 (0.82–0.95)	<0.001
Presence of NAFLD (yes/no)	0.83 (0.65–1.07)	0.147			1.48 (1.11–1.97)	0.008		
Presence of NASH (yes/no)	3.02 (1.90–4.82)	<0.001	2.53 (1.52–4.21)	<0.001	1.95 (1.25–3.05)	0.003	1.83 (1.09–3.09)	0.023

Fibrosis was assessed according to Scheuer’s scoring system, in which significant fibrosis was S ≥ 2 and severe fibrosis was S ≥ 3.

Overweight, body mass index ≥ 23 kg/m^2^; TB, total bilirubin; ALT, alanine aminotransferase; AST, aspartate aminotransferase; HBV, hepatitis B virus; HCC, hepatocellular carcinoma; NAFLD, non-alcoholic fatty liver disease; NAFL, non-alcoholic fatty liver; NASH, non-alcoholic steatohepatitis.

### Correlation Between Histological Changes and Severity of Liver Fibrosis

**Figure 2:**
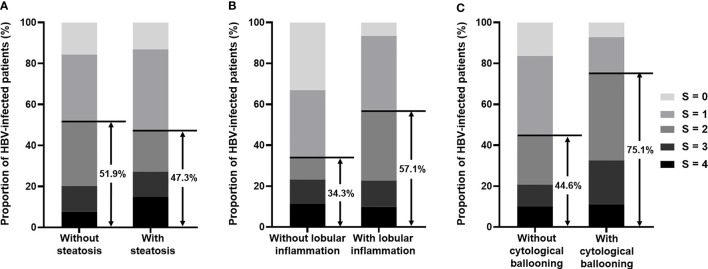
**(A)** Bar chart illustrates the proportion of fibrosis stage in HBV-infected patients with or without steatosis. Among patients without steatosis, 51.9% had significant fibrosis (S ≥ 2), and among patients with steatosis, 47.3% had significant fibrosis (S ≥ 2). **(B)** Bar chart illustrates the proportion of fibrosis stage in HBV-infected patients with or without lobular inflammation. Among patients without lobular inflammation, 34.3% had significant fibrosis (S ≥ 2), and among patients with lobular inflammation, 57.1% had significant fibrosis (S ≥ 2). **(C)** Bar chart illustrates the proportion of fibrosis stage in HBV-infected patients with or without cytological ballooning. Among patients without cytological ballooning, 44.6% had significant fibrosis (S ≥ 2), and among patients with cytological ballooning, 75.1% had significant fibrosis (S ≥ 2). HBV, hepatitis B virus.

Based on the significant association of significant/severe fibrosis with the presence of NASH, we further compared the distribution of fibrosis stages based on Scheuer’s scoring system between patients with and without steatosis, lobular inflammation, or cytological ballooning and found that the proportion of significant fibrosis was significantly different between patient with or without lobular inflammation (57.1% vs. 34.3%, *p* < 0.001) and patients with or without cytological ballooning (75.1% vs. 44.6%, *p* < 0.001) (**Figure 2**). The proportion of significant fibrosis showed no difference between patients with or without steatosis (47.3% vs. 51.9%, *p* = 0.146) (**Figure 2**). According to spearman correlation analyses, both the degree of lobular inflammation and degree of cytological ballooning were significantly associated with fibrosis stage (both *p* < 0.001) (**Table 3**). The presence of steatosis, presence of moderate-to-severe steatosis, and degree of steatosis were not related to the fibrosis stage (all *p* > 0.05).

**Table 3:** Spearman’s correlation analysis between three histological changes and severity of fibrosis.

Variable	Fibrosis stage	
	*rho*	*p*-Value
Presence of steatosis (yes/no)	0.025	0.419
Moderate-to-severe steatosis (yes/no)	0.024	0.436
Degree of steatosis	0.026	0.386
Presence of lobular inflammation (yes/no)	0.208	<0.001
Degree of lobular inflammation	0.209	<0.001
Presence of cytological ballooning (yes/no)	0.199	<0.001
Degree of cytological ballooning	0.194	<0.001

Fibrosis was assessed according to Scheuer’s scoring system.

### Factors Associated With Non-Alcoholic Steatohepatitis-Related Histological Changes

Correlation analyses of metabolic and viral factors and NASH-related histological changes were performed according to Spearman’s method (**Table 4**). It was demonstrated that metabolic factors (including BMI, diabetes, and hypertension) were mildly correlated with the degree of steatosis or cytological ballooning but not lobular inflammation. Among viral factors, HBV DNA but not HBsAg or HBeAg status was associated with lobular inflammation. Clinical factors related to lobular inflammation only included ALT and AST.

**Table 4:** Correlation analysis between metabolic, virological factors, and NASH-related histological changes.

Variable	Degree of steatosis		Degree of lobular inflammation		Degree of cytological ballooning	
	*rho*	*p*-Value	*rho*	*p*-Value	*rho*	*p*-Value
Diabetes (yes/no)	0.079	0.009	0.022	0.475	0.064	0.037
Hypertension (yes/no)	0.078	0.011	0.041	0.180	0.137	<0.001
BMI (kg/m^2^)	0.315	<0.001	0.052	0.095	0.189	<0.001
HBsAg (log IU/ml)	0.015	0.716	0.046	0.249	−0.043	0.286
HBeAg-positive (yes/no)	−0.011	0.720	0.060	0.052	−0.129	<0.001
HBV DNA (log IU/ml)	−0.042	0.182	0.124	<0.001	−0.097	0.002

BMI, body mass index; HBsAg, hepatitis B surface antigen; NASH, non-alcoholic steatohepatitis; HBeAg, hepatitis B e-antigen; HBV, hepatitis B virus.

### Subgroup Analysis of the Association Between Non-Alcoholic Steatohepatitis and Significant Fibrosis

To further analyze the association between NASH and significant fibrosis in different populations, we performed univariable analyses and multivariable analyses in subgroups (HBeAg-positive, HBeAg-negative, ALT ≤ ULN, ALT > ULN, and BMI < 23 kg/m^2^). The results showed that NASH was independently related to significant fibrosis in four subgroups (*p* = 0.004 for HBeAg-positive; *p* = 0.008 for HBeAg-negative; *p* = 0.007 for ALT ≤ ULN; *p* = 0.038 for ALT > ULN) (**Tables 5** and **6**).

**Table 5:** Univariable analyses and multivariable analyses of factors associated with significant fibrosis in HBeAg-positive and HBeAg-positive subgroups.

Variable	HBeAg-positive (n = 582)				HBeAg-positive (n = 464)			
	Univariable analysis		Multivariable analysis		Univariable analysis		Multivariable analysis	
	OR (95% CI)	*p*-Value	OR (95% CI)	*p*-Value	OR (95% CI)	*p*-Value	OR (95% CI)	*p*-Value
Age (years)	1.03 (1.03–1.05)	<0.001			1.01 (0.99–1.03)	0.200		
Male (yes/no)	1.12 (0.80–1.57)	0.506			1.25 (0.85–1.84)	0.253		
Diabetes (yes/no)	1.36 (0.36–5.13)	0.647			4.12 (0.48–35.57)	0.198		
Hypertension (yes/no)	3.31 (0.66–16.53)	0.145			0.96 (0.42–2.19)	0.919		
Overweight (yes/no)	1.86 (1.33–2.61)	<0.001	1.76 (1.20–2.58)	0.004	1.59 (1.09–2.31)	0.016		
Family HCC history (yes/no)	1.17 (0.69–1.98)	0.557			1.22 (0.71–2.11)	0.475		
Platelets (10^9^/L)	0.99 (0.99–0.99)	<0.001	0.99 (0.99–0.99)	<0.001	0.99 (0.99–1.00)	<0.001	0.99 (0.99–1.00)	<0.001
Albumin (g/L)	0.96 (0.92–1.00)	0.077			1.02 (0.97–1.08)	0.357		
TB (μmol/L)	1.01 (0.99–1.02)	0.404			1.00 (0.98–1.03)	0.957		
ALT (IU/L)	1.00 (1.00–1.00)	0.058			1.00 (1.00–1.01)	0.522		
AST (IU/L)	1.00 (1.00–1.00)	0.428			1.01 (1.00–1.02)	0.037		
HBsAg (log IU/ml)	0.59 (0.45–0.78)	<0.001			1.25 (0.96–1.62)	0.099		
HBV DNA (log IU/ml)	0.73 (0.65–0.82)	<0.001	0.78 (0.68–0.89)	<0.001	1.12 (0.97–1.29)	0.130		
Presence of NAFLD	1.07 (0.77–1.51)	0.678			0.61 (0.42–0.89)	0.010		
Presence of NASH	3.64 (1.69–7.87)	0.001	3.38 (1.47–7.79)	0.004	2.49 (1.36–4.56)	0.003	2.32 (1.24–4.34)	0.008

Fibrosis was assessed according to Scheuer’s scoring system, in which significant fibrosis was S ≥ 2.

Overweight, BMI ≥ 23 kg/m^2^; TB, total bilirubin; ALT, alanine aminotransferase; AST, aspartate aminotransferase; HBsAg, hepatitis B surface antigen; HBeAg, hepatitis B e-antigen; OR, odds ratio; HCC, hepatocellular carcinoma; HBV, hepatitis B virus.

**Table 6:** Univariable analyses and multivariable analyses of factors associated with significant fibrosis among patients with normal ALT or abnormal ALT.

Variable	ALT ≤ ULN (n = 347)				ALT > ULN (n = 669)			
	Univariable analysis		Multivariable analysis		Univariable analysis		Multivariable analysis	
	OR (95% CI)	*p*-Value	OR (95% CI)	*p*-Value	OR (95% CI)	*p*-Value	OR (95% CI)	*p*-Value
Age (years)	1.02 (1.00–1.04)	0.019			1.04 (1.02–1.06)	<0.001	1.02 (1.00–1.04)	0.042
Male (yes/no)	1.13 (0.79–1.61)	0.614			1.18 (0.81–1.72)	0.403		
Diabetes (yes/no)	5.17 (0.60–44.56)	0.135			0.93 (0.23–3.74)	0.914		
Hypertension (yes/no)	1.14 (0.45–2.84)	0.787			2.12 (0.65–6.98)	0.215		
Overweight (yes/no)	1.90 (1.31–2.75)	<0.001	1.76 (1.20–2.58)	0.007	1.59 (1.09–2.31)	0.007		
Family HCC history (yes/no)	1.17 (0.66–2.08)	0.601			1.09 (0.66–1.82)	0.738		
Platelets (10^9^/L)	0.99 (0.99–1.00)	<0.001	0.99 (0.99–0.99)	<0.001	0.99 (0.98–0.99)	<0.001	0.99 (0.98–0.99)	<0.001
Albumin (g/L)	1.02 (0.98–1.07)	0.359			0.97 (0.92–1.01)	0.140		
TB (μmol/L)	1.00 (0.98–1.02)	0.739			1.01 (0.99–1.02)	0.452		
ALT (IU/L)	1.02 (1.00–1.05)	0.081			1.00 (1.00–1.00)	0.006		
AST (IU/L)	1.05 (1.02–1.07)	<0.001	1.04 (1.01–1.07)	0.006	1.00 (1.00–1.00)	0.327		
HBsAg (log IU/ml)	0.95 (0.76–1.18)	0.629			0.65 (0.50–0.84)	<0.001		
HBV DNA (log IU/ml)	0.91 (0.83–1.00)	0.060			0.78 (0.71–0.87)	<0.001	0.83 (0.74–0.93)	0.001
Presence of NAFLD	0.82 (0.56–1.18)	0.304			0.80 (0.57–1.13)	0.211		
Presence of NASH	3.14 (1.54–6.40)	0.002	2.86 (1.34–6.10)	0.007	2.76 (1.46–5.23)	0.002	2.09 (1.04–4.18)	0.038

Fibrosis was assessed according to Scheuer’s scoring system, in which significant fibrosis was S ≥ 2.

Overweight, BMI ≥ 23 kg/m^2^; TB, total bilirubin; ALT, alanine aminotransferase; AST, aspartate aminotransferase; ULN, upper limit of normal; HBsAg, hepatitis B surface antigen; HCC, hepatocellular carcinoma; NAFLD, non-alcoholic fatty liver disease; NASH, non-alcoholic steatohepatitis; HBV, hepatitis B virus.

Moreover, 512 (47.4%) patients were classified as lean CHB, among whom 122 (23.8%) and 18 (3.5%) patients had concomitant NAFLD or NASH, respectively according to the FLIP algorithm (**Supplementary Figure 2**). Patient characteristics and pathological features according to the presence of NAFLD or NASH are enumerated in **Supplementary Table 3**. Similar to the results in the entire cohort, significant fibrosis was more frequently presented in lean patients with lobular inflammation (52.7% vs. 27.9%, *p* < 0.01), or with cytological ballooning (68.9% vs. 41.5%, *p* < 0.01) but not with steatosis (47.3% vs. 53.6%, *p* = 0.171) than those without (**Supplementary Figure 3**). The OR of significant fibrosis and severe fibrosis in this specific population was 2.55 (95% CI 0.94–6.91, *p* = 0.065) and 3.06 (95% CI 1.15–8.13, *p* = 0.025), respectively.

## Discussion

With the increasing prevalence of NAFLD, more and more attention is focused on the effects of NAFLD or NASH on disease progression in CHB infection. This study retrospectively investigated the biopsy-proven NAFLD/NASH as per the FLIP algorithm (Nascimbeni et al., 2020) in consecutive antiviral-naïve CHB patients with the largest sample size up to date (Thomopoulos et al., 2006; Bondini et al., 2007; Zheng et al., 2010; Charatcharoenwitthaya et al., 2017; Shen et al., 2019). We identified 37.4% of patients with co-existing NAFLD (intrahepatic steatosis ≥ 5%) and 24.0% of patients who have concomitant NAFLD were diagnosed with NASH. The incidence of NAFLD in CHB patients is controversial. It has been reported that CHB patients had a lower incidence of NAFLD (Zhong et al., 2018). In Zhong’s study, NAFLD was diagnosed by abdominal ultrasonography, which is not sensitive to mild steatosis. Other studies reported that the prevalence of NAFLD detected by liver biopsy was 37.6% (Shen et al., 2019) or 38.3% (Charatcharoenwitthaya et al., 2017), which is similar to our results. What is more, lobular inflammation and cytological ballooning had significant effects on the severity of liver fibrosis, but the presence of steatosis did not, which reinforced the conclusion that inflammation activity and hepatocyte lesion caused by excessive intrahepatic steatosis deposition are chief culprits responsible for fibrosis progression in CHB patients (Machado and Diehl, 2016). Among patients with normal ALT, the occurrence of NASH is strongly associated with significant fibrosis. Such effect was independent of HBeAg status. Exactly as a previous study shows that the proportion of NASH patients with normal ALT in overall NASH patients was 19% (95% CI 13%–27%) (Ma et al., 2020), ALT is not sensitive in predicting the presence of NASH. The hidden fibrotic influence of NASH among patients with normal liver function has always been neglected.

Meanwhile, we found 484 (44.8%) patients with no steatosis but with lobular inflammation in this study. It could be related to HBV itself. These HBV patients might be categorized as having HBV-related lobular activity. Histological lesions such as spotty lobular inflammation are common in CHB and, therefore, are not specific for steatohepatitis (Mani and Kleiner, 2009). It is exceedingly difficult to affirm the overlap of viral hepatitis and steatohepatitis in histological activity. Peng et al. found steatosis to be more frequent in CHB than in the general population, and they hypothesize that this may be due to metabolic factors or the ability of HBV to indirectly facilitate the development of steatosis (Peng et al., 2008). On the other hand, some studies demonstrated that HBV infection may exacerbate metabolic dysregulation in NAFLD in animal and cell models. Kim et al. found that HBV participates in the activation of SREBP1 and peroxisome proliferator-activated receptor-γ (PPAR-γ) (Kim et al., 2007; Kim et al., 2008). HBV infection may exacerbate metabolic dysregulation in NAFLD as suggested by previous studies based on animal models (Zhang et al., 2020). The interaction between HBV infection and steatosis still needs to be fully elucidated in the future. On the other hand, there are different opinions about concurrent NAFLD downregulating the viral titer in CHB patients. A previous study in Hong Kong reported the inverse relationship between hepatic steatosis (CAP ≥ 222 dB/m) and hepatitis B viremia in treatment-naïve CHB patients by multivariable logistic analysis (*p* = 0.041; OR, 0.859, 95% CI 0.743–0.994) (Hui et al., 2018). However, Charatcharoenwitthaya et al. thought that there is no association between HBV DNA and histological-proven steatosis (*p* = 0.599) in the Asian population according to a univariable logistic analysis (Charatcharoenwitthaya et al., 2017). Our data verified the result and demonstrated that the presence of steatosis was correlated with metabolic factors (including BMI, diabetes, and hypertension), rather than HBV DNA level (*p* = 0.310). The bidirectional correlation of CHB and NAFLD is complicated and is worthy of future study.

Our findings are of important clinical implications. First, we reproved that liver fibrosis in CHB patients concurrent with NASH is severer than in CHB patients without NASH. Attention should be focused on routine assessment for concomitance of NAFLD/NASH apart from management of HBV infection. Second, a wider indication for liver biopsies should be considered among patients who are highly suspected to have concomitant steatohepatitis. At present, no available non-invasive test (NIT) has acceptable accuracy, and liver biopsy remains the reference standard for the diagnosis of NASH (European Association for the Study of the Liver. Electronic address et al., 2021). Since the presence of NASH was associated with fibrosis progression among patients with normal ALT, more concerns are needed for patients with normal ALT who were universally thought to have no or mild histological changes in the liver. As the latest EASL Clinical Practice Guidelines pointed out, automatic calculation and systematic reporting of simple non-invasive fibrosis tests such as Fibrosis-4 (FIB-4) and routine detection of liver stiffness by transient elastography are beneficial to screening for liver fibrosis currently (European Association for the Study of the Liver. Electronic address et al., 2021). Lastly, early recognition and management of NAFLD and its associated metabolic disorders in CHB patients may bring a beneficial effect on their long-term outcomes. To CHB patients with NASH, the treatment strategy should include treatment of metabolic dysfunction and drug therapies targeting NASH when available, besides weight loss and lifestyle modifications.

Our study is limited by the retrospective design. The lack of data, including dyslipidemia, waist–hip ratios, and blood pressure levels, precludes us from making a more convincing conclusion. Moreover, the evaluation of the SAF score was not cross-validated by two or more pathologists, and complete pathological assessment including assessment of portal inflammation and lack of confluent necrosis might strengthen and enrich our conclusions. A well-developed prospective cohort study to further investigate the influence of NASH in fibrosis progression and long-term outcomes among CHB patients will be needed.

In conclusion, NAFLD is common in chronic HBV-infected patients, while NASH is associated with an increased risk of significant or severe fibrosis, suggesting a contributive effect on the pathogenesis of disease progression in CHB patients. More concern should be paid to the assessment of steatohepatitis and overweight besides managing CHB. Liver biopsies are recommended for CHB patients who are highly suspected of having co-existing NASH. Efforts on ameliorating steatohepatitis by current approaches available are urgent in CHB patients who have concomitant NASH.

## Data Availability Statement

The raw data supporting the conclusions of this article will be made available by the authors, without undue reservation.

## Ethics Statement

The studies involving human participants were reviewed and approved by Ruijin Hospital Ethics Committee, Shanghai Jiao Tong University School of Medicine. Written informed consent for participation was not required for this study in accordance with the national legislation and the institutional requirements.

## Author Contributions

QX, ZC, and YH conceived and designed the study. YH, ZC, QYG, RL, WW, SG, ZS, LC, and GZ collected the data. YH conducted the analysis and drafted the manuscript. QX, ZC, GZ, HW, and WC interpreted the data. QX, ZC, and QYG critically revised the manuscript. All authors contributed to the article and approved the submitted version.

## Funding

This work was supported by the National Natural Science Foundation of China (No. 81570535 and No. 81770587), the Shanghai Three-Year Plan of the Clinical Skills and Innovations (16CR1002A), the Shanghai Municipal Key Clinical Specialty (shslczdzk01103), and the Shanghai Three-Year Plan of the Key Subjects Construction in Public Health-Infectious Diseases and Pathogenic Microorganism (15GWZK0102).

## Conflict of Interest

The authors declare that the research was conducted in the absence of any commercial or financial relationships that could be construed as a potential conflict of interest.

## Publisher’s Note

All claims expressed in this article are solely those of the authors and do not necessarily represent those of their affiliated organizations, or those of the publisher, the editors and the reviewers. Any product that may be evaluated in this article, or claim that may be made by its manufacturer, is not guaranteed or endorsed by the publisher.

## Supplementary Material

Supplementary Figure 1 Patients recruitment. Patients with NASH was diagnosed as SAF Score; HCC: hepatocellular carcinoma; excessive alcohol consumption: defined as alcohol intake ≥20 g per day for men and ≥10 g per day for women; poor quality liver samples: defined as liver sample less than 10mm in length or containing less than six portal triads.

Supplementary Figure 2
**(A)** FLIP algorithmic tree in lean population. A total of 512 patients with treatment naïve chronic hepatitis B with BMI ≤23 kg/m^2^ were classified into no NAFLD, NAFL, NASH or severe NASH according to the FLIP algorithmic pathways based on the grade of steatosis (Panel a, light yellow shade), grade of Ballooning (Panel a, light blue shade) and grade of lobular inflammation (Panel a, pink shade). Number of patients and the proportion among each category were presented as number (%). **(B)** Pie chart to illustrate the proportion of lean patients (BMI ≤23 kg/m2) with specific diagnosis. Among all 512 patients, 76.2% had no NAFLD and the remaining 23.8% had NAFLD, among which, 85.3% had NAFL and 14.8% had NASH. FLIP, fatty liver inhibition of progression; NAFLD, non-alcoholic fatty liver disease; NAFL, non-alcoholic fatty liver; NASH, non-alcoholic steatosis hepatitis.

Supplementary Figure 3
**(A)** Bar chart to illustrate the proportion of fibrosis stage in HBV-infected patients with or without steatosis. 53.6% had significant fibrosis (S≥2) among patients without steatosis and 47.3% had significant fibrosis (S≥2) among patients with steatosis. **(B)** Bar chart to illustrate the proportion of fibrosis stage in HBV-infected patients with or without lobular inflammation. 27.9% had significant fibrosis (S≥2) among patients without lobular inflammation and 52.7% had significant fibrosis (S≥2) among patients with lobular inflammation. **(C)** Bar chart to illustrate the proportion of fibrosis stage in HBV-infected patients with or without cytological ballooning. 41.5% had significant fibrosis (S≥2) among patients without cytological ballooning and 68.9% had significant fibrosis (S≥2) among patients with cytological ballooning.
