# Supplementary figures and images for: Application of Fatty Liver Inhibition of Progression Algorithm and Steatosis, Activity, and Fibrosis Score to Assess the Impact of Non-Alcoholic Fatty Liver on Untreated Chronic Hepatitis B Patients
Source: Front Cell Infect Microbiol. 2022 Jan 17;11:733348. doi: 10.3389/fcimb.2021.733348 (PMC8801606; doi:10.3389/fcimb.2021.733348)

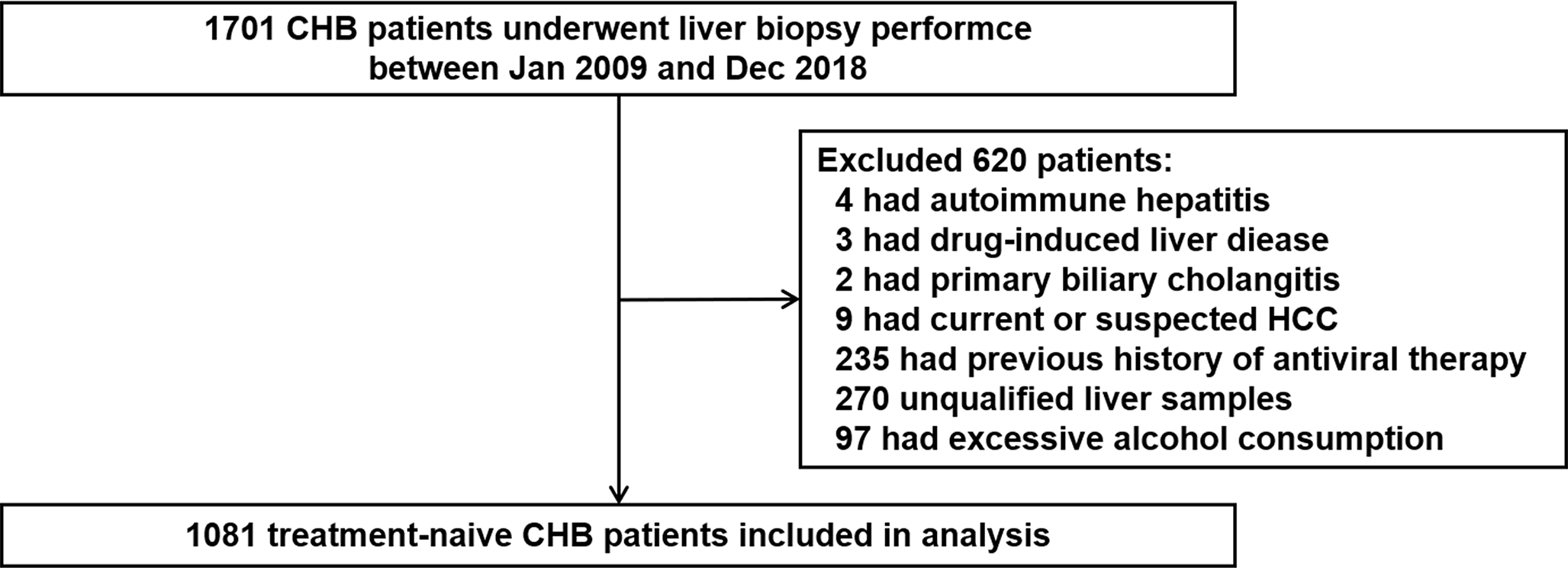

Supplement: Supplementary Figure 1 — Patients recruitment. Patients with NASH was diagnosed as SAF Score; HCC: hepatocellular carcinoma; excessive alcohol consumption: defined as alcohol intake ≥20 g per day for men and ≥10 g per day for women; poor quality liver samples: defined as liver sample less than 10mm in length or containing less than six portal triads. [file Image_1.tif]

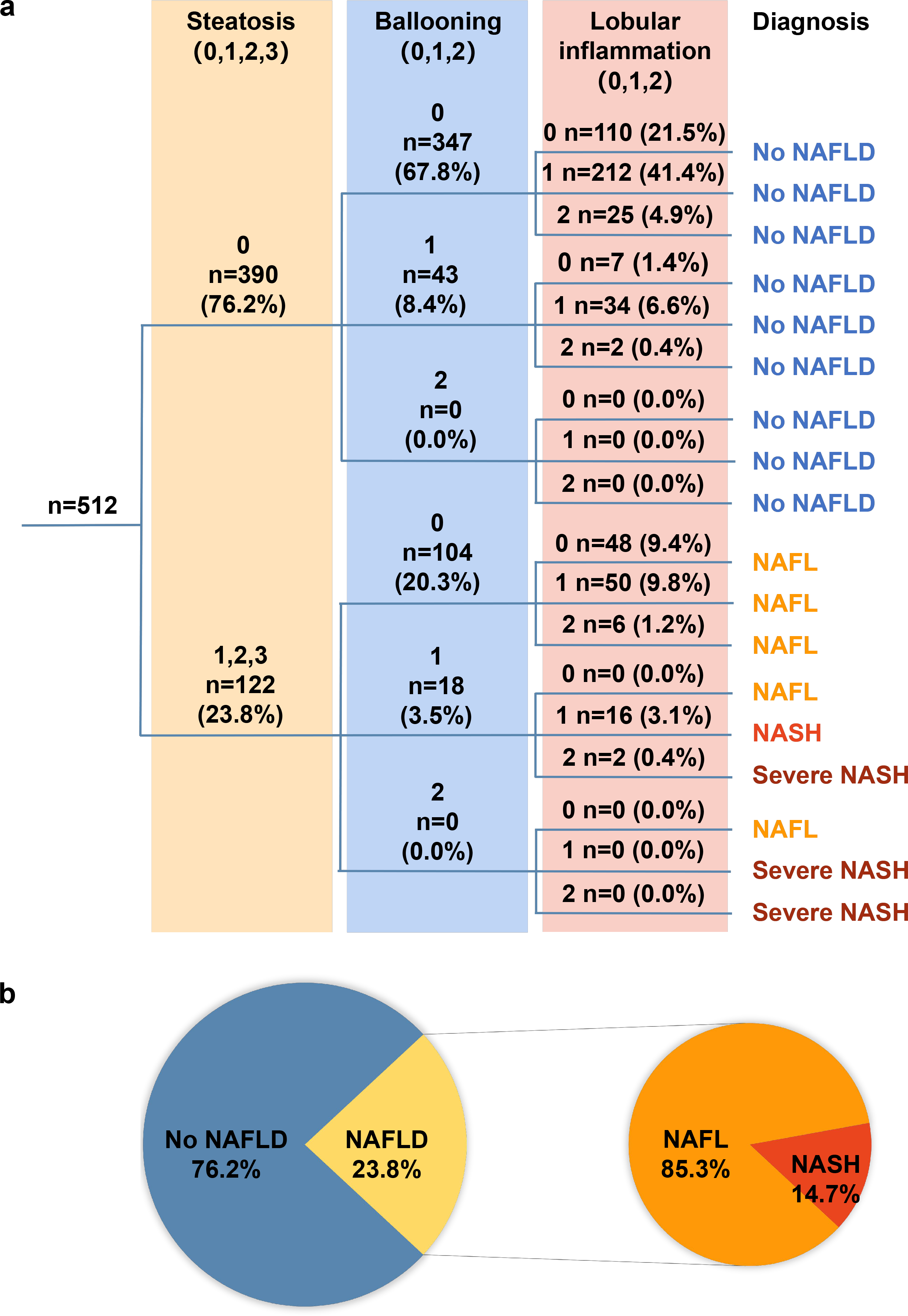

Supplement: Supplementary Figure 2 — (A) FLIP algorithmic tree in lean population. A total of 512 patients with treatment naïve chronic hepatitis B with BMI ≤23 kg/m2 were classified into no NAFLD, NAFL, NASH or severe NASH according to the FLIP algorithmic pathways based on the grade of steatosis (Panel a, light yellow shade), grade of Ballooning (Panel a, light blue shade) and grade of lobular inflammation (Panel a, pink shade). Number of patients and the proportion among each category were presented as number (%). (B) Pie chart to illustrate the proportion of lean patients (BMI ≤23 kg/m2) with specific diagnosis. Among all 512 patients, 76.2% had no NAFLD and the remaining 23.8% had NAFLD, among which, 85.3% had NAFL and 14.8% had NASH. FLIP, fatty liver inhibition of progression; NAFLD, non-alcoholic fatty liver disease; NAFL, non-alcoholic fatty liver; NASH, non-alcoholic steatosis hepatitis. [file Image_2.tif]

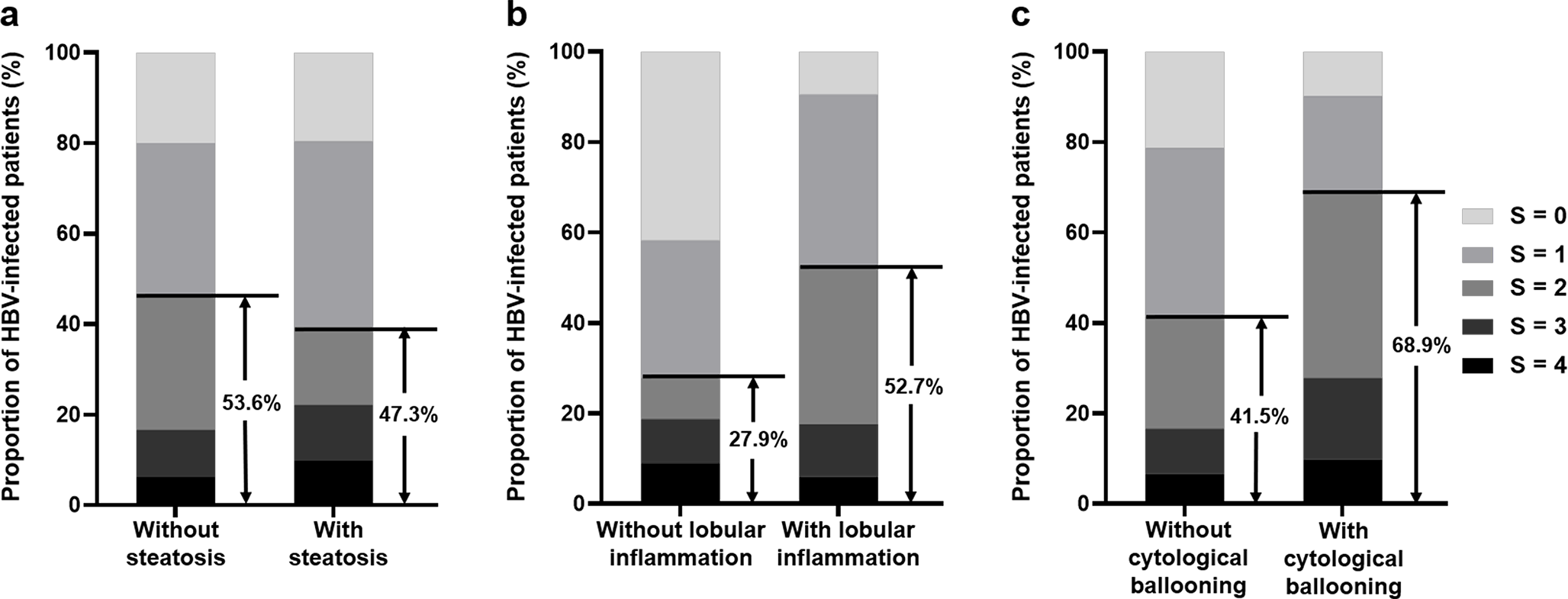

Supplement: Supplementary Figure 3 — (A) Bar chart to illustrate the proportion of fibrosis stage in HBV-infected patients with or without steatosis. 53.6% had significant fibrosis (S≥2) among patients without steatosis and 47.3% had significant fibrosis (S≥2) among patients with steatosis. (B) Bar chart to illustrate the proportion of fibrosis stage in HBV-infected patients with or without lobular inflammation. 27.9% had significant fibrosis (S≥2) among patients without lobular inflammation and 52.7% had significant fibrosis (S≥2) among patients with lobular inflammation. (C) Bar chart to illustrate the proportion of fibrosis stage in HBV-infected patients with or without cytological ballooning. 41.5% had significant fibrosis (S≥2) among patients without cytological ballooning and 68.9% had significant fibrosis (S≥2) among patients with cytological ballooning. [file Image_3.tif]
