# Supplementary material for: Application of Fatty Liver Inhibition of Progression Algorithm and Steatosis, Activity, and Fibrosis Score to Assess the Impact of Non-Alcoholic Fatty Liver on Untreated Chronic Hepatitis B Patients
Source: Front Cell Infect Microbiol. 2022 Jan 17;11:733348. doi: 10.3389/fcimb.2021.733348 (PMC8801606; doi:10.3389/fcimb.2021.733348)
Supplement: Supplementary file 4 [file DataSheet_1.docx]

Supplementary Table 1. Demographic, clinical, biochemical characteristics and liver pathological features of lean patient.

| Variable | Lean CHB (n=512) | Lean CHB without NAFLD (n=390) | NAFL (n=104) | NASH (n=18) | *P*-value^1^ |
| --- | --- | --- | --- | --- | --- |
| Age (yrs) | 36 (29-45) | 36 (29-44) | 36 (30-45) | 40 (33-51) | 0.346 |
| Male (n, %) | 280 (54.7%) | 195 (50.0%) | 71 (68.3%) | 14 (77.8%) | <0.001 |
| BMI (kg/m^2^) | 20.76 (19.6-22.0) | 20.65 (19.5-21.9) | 20.80 (20.0-22.2) | 21.49 (20.3-22.3) | 0.030 |
| Diabetes (n, %) | 5 (1.0%) | 1 (0.3%) | 2 (1.9%) | 2 (11.1%) | <0.001 |
| Arterial hypertension (n, %) | 10 (2.0%) | 8 (2.1%) | 1 (1.0%) | 1 (5.6%) | 0.781 |
| Family HCC history (n, %) | 48 (9.4%) | 36 (9.2%) | 11 (10.6%) | 1 (5.6%) | .971 |
| Platelets (10^9^/L) | 173 (141-205) | 174 (141-205) | 171 (141-205) | 177 (141-212) | 0.962 |
| Alb (g/L) | 44 (41-46) | 44 (41-46) | 43 (40-46) | 45 (42-47) | 0.177 |
| TB (μmol/L) | 16 (12-20) | 15 (12-20) | 17 (13-21.5) | 15 (10-21) | 0.062 |
| ALT (IU/L) | 39 (25-71) | 37 (25-68) | 47 (30-92) | 39 (23-45) | 0.012 |
| ALT ＞ 40 IU/L (n, %) | 232 (47.6%) | 168 (45.2%) | 57 (58.2%) | 7 (41.2%) | 0.155 |
| AST (IU/L) | 33 (25-49) | 33 (24-48) | 40 (27-62) | 29 (27-36) | 0.030 |
| HBsAg (log IU/mL) | 3.59 (3.03-4.39) | 3.53 (2.98-4.22) | 3.60 (3.14-4.67) | 3.88 (3.36-4.31) | 0.132 |
| HBeAg-positive (n, %) | 276 (55.5%) | 210 (55.3%) | 59 (59.6%) | 7 (38.9%) | 0.733 |
| HBV DNA (log IU/mL) | 5.68 (3.82-7.37) | 5.58 (3.85-7.42) | 6.14 (3.91-7.41) | 5.13 (3.17-6.33) | 0.262 |
| Significant fibrosis (n, %) | 229 (44.7%) | 181 (46.4%) | 36 (34.6%) | 12 (66.7%) | 0.654 |
| Severe fibrosis (n, %) | 92 (18.0%) | 65 (16.7%) | 20 (19.2%) | 7 (38.9%) | 0.050 |
| Steatosis (n, %) | | | | | |
| 0 - < 5% | 390 (76.2%) | 390 (100.0%) | 0 (0%) | 0 (0%) | <0.001 |
| 1 - 5-33% | 99 (19.3%) | 0 (0%) | 84 (80.8%) | 15 (83.3%) |  |
| 2 - 33-66% | 22 (4.3%) | 0 (0%) | 19 (18.3%) | 3 (16.7%) |  |
| 3 - ≥ 66% | 1 (0.2%) | 0 (0%) | 1 (1.0%) | 0 (0.0%) |  |
| Lobular inflammation (n, %) | | | | | |
| 0 - No foci | 165 (32.2%) | 117 (30.0%) | 48 (46.2%) | 0 (0%) | 0.715 |
| 1 - < 2 foci | 312 (60.9%) | 246 (63.1%) | 50 (48.1%) | 16 (88.9%) |  |
| 2 -  ≥ 2 foci | 35 (6.8%) | 27 (6.9%) | 6 (5.8%) | 2 (11.1%) |  |
| Cytological ballooning (n, %) | | | | | |
| 0 - None | 451 (88.1%) | 347 (89.0%) | 104 (100.0%) | 0 (0%) | <0.001 |
| 1 - Few | 61 (11.9%) | 43 (11.0%) | 0 (0.0%) | 18 (100.0%) |  |
| 2 - Many | 0 (0.0%) | 0 (0.0%) | 0 (0.0%) | 0 (0.0%) |  |

Data are presented as mean ± SD, median (IQR) or number of patients (%).

^1^P-value for comparison among 3 groups of no NAFLD, NAFL and NASH.

Abbreviations: BMI, body mass index; TB, total bilirubin; ALT, alanine aminotransferase; AST, aspartate aminotransferase; ULN, upper limit of normal; HBsAg, Hepatitis B surface antigen.
